# Interfacial Engineering of Co_3_O_4_@MXene for Superior Charge Storage: A Route to High-Capacitance Supercapacitors

**DOI:** 10.3390/mi16121313

**Published:** 2025-11-24

**Authors:** Qasim Raza, Sooman Lim

**Affiliations:** Graduate School of Flexible and Printable Electronics, LANL-JBNU Engineering Institute-Korea, Jeonbuk National University, Jeonju 54896, Republic of Korea; qasimraza158@gmail.com

**Keywords:** nanocomposite, pseudo capacitance, electrochemical energy storage, supercapacitors, charge–discharge kinetics

## Abstract

We report an interracially engineered Co_3_O_4_@Ti_3_C_2_T_x_ MXene hybrid as a high-rate charge-storage electrode. Low-temperature assembly under inert conditions preserves the MXene carbide while anchoring nanocrystalline Co_3_O_4_ on conductive, ion-permeable sheets. XRD and FTIR confirm the structural integrity of MXene without TiO_2_ formation. Electrochemically, cyclic voltammetry, Dunn analysis, and galvanostatic tests reveal mixed storage with a dominant pseudocapacitive contribution, while EIS fitting shows reduced charge-transfer resistance for the hybrid compared with either parent. Within a 0.6 V window in 2 M KOH, the composite delivers high specific charge and excellent rate retention, attributable to shortened diffusion paths and fast electron transport at the oxide–MXene interface. These results establish Co_3_O_4_@MXene as a robust, mechanism-consistent platform for high-power supercapacitors.

## 1. Introduction

The growing global energy demand and the integration of renewable energy sources have created an urgent need for reliable, sustainable, and high-performance energy storage technologies [1,2]. Supercapacitors (SCs) have attracted significant attention because of their high power density, rapid charge–discharge rates, and long cycling stability compared to conventional batteries [3,4]. However, their relatively low energy density limits their practical use in applications that require both high power and high energy output. Therefore, considerable research efforts are devoted to the rational design of advanced electrode materials that can combine high capacitance, good rate capability, and excellent durability.

Transition metal oxides (TMOs) are among the most studied pseudocapacitive materials due to their multiple valence states and high theoretical capacitance [5,6,7]. Cobalt oxides, including Co_3_O_4_ and Co_2_O_3_, have been highlighted because of their rich redox chemistry, environmental benignity, and relatively low cost [8,9]. For instance, Co_3_O_4_ nanosheets have been reported to deliver specific capacitances exceeding 1200 F g^−1^ at 1 A g^−1^, though their performance suffers from poor electrical conductivity and structural degradation upon cycling [10]. Similarly, nanostructured Co_2_O_3_ electrodes show promising pseudocapacitive behavior but still face limitations associated with sluggish charge transport and low cycling stability [11].

On the other hand, MXenes—a new class of two-dimensional transition metal carbides and nitrides (M_n+1_X_n_T_x_)—have recently emerged as promising electrode candidates owing to their metallic-level conductivity, hydrophilic surfaces, and tunable interlayer spacing [12,13]. Ti_3_C_2_T_x_ MXene, the most studied member, can achieve volumetric capacitances above 1500 F cm^−3^ and cycle lives exceeding 10,000 cycles [14]. However, pure MXene electrodes mainly rely on electric double-layer capacitance, which limits their energy density. In addition, restacking of MXene nanosheets during cycling reduces ion accessibility and active surface area [15].

MXenes are two-dimensional transition-metal carbides and nitrides with the general formula M_n+1_X_n_T_x_, where M is an early transition metal, X is C and/or N, and T_x_ denotes surface terminations such as –O, –OH, and –F introduced during etching. In Ti_3_C_2_T_x_, selective removal of Al from Ti_3_AlC_2_ produces stacked layers that can be delaminated into few-layer sheets with metallic-level conductivity and hydrophilic surfaces. The (002) reflection at low 2θ in XRD reports the interlayer spacing, which increases upon intercalation and surface termination, while the in-plane order is preserved. These structural features enable rapid ion intercalation and high volumetric capacitance, and they provide a conductive scaffold for redox-active nanoparticles [16].

To address these limitations, hybridization of TMOs with MXenes has been proposed as an effective strategy. The TMOs contribute Faradaic pseudocapacitance, while MXenes act as conductive scaffolds that accelerate electron/ion transport and suppress agglomeration [17]. As MnO_2_@MXene composites have shown capacitances above 700 F g^−1^ with improved cycling stability compared to pristine MnO_2_ [18]. Similarly, NiO/MXene hybrids demonstrated enhanced capacitance and reduced internal resistance compared with standalone NiO electrodes [19]. In the case of cobalt oxides, Co_3_O_4_/MXene composites have been reported to deliver capacitances over 1500 F g^−1^, far higher than either component alone [20]. These studies highlight the synergistic benefits of combining TMOs with MXenes.

In this study, we synthesized a Co_3_O_4_@MXene nanocomposite and systematically investigated its structural, chemical, and electrochemical properties. The results demonstrate that the hybrid electrode exhibits superior capacitive performance compared to pristine Co_3_O_4_ and MXene, highlighting its potential as a next-generation electrode material for high-performance supercapacitors.

## 2. Experimental Section

### 2.1. Materials

MXene precursor MAX powder (Ti_3_AlC_2_, 99%), hydrochloric acid (HCl, 37%), lithium fluoride (LiF, 99%), potassium hydroxide (KOH, 98%), ammonia, and Nafion (5 wt%) were purchased from Sigma Aldrich (St. Louis, MO, USA). Distilled water (H_2_O), ethanol (C_2_H_5_OH), and cobalt nitrate were purchased from science 4u. All chemicals were used as received without any additional purification.

### 2.2. Synthesis of Pristine Co_3_O_4_ via Hydrothermal Method

Pristine cobalt oxide (Co_3_O_4_) nanoparticles were synthesized using a hydrothermal method with cobalt nitrate as the precursor. Initially, cobalt nitrate was dissolved in distilled water to form a clear cobalt ion solution. Ammonia solution was added dropwise under continuous stirring to adjust the pH and induce precipitation of cobalt hydroxide species. The resulting mixture was transferred into a Teflon-lined stainless-steel autoclave and subjected to hydrothermal treatment at 120 °C for 12 h to facilitate controlled nucleation and growth of cobalt oxide nanoparticles. After the hydrothermal process, obtained precipitate was collected by centrifugation and thoroughly washed with distilled water to remove residual ions and impurities. The washed solid was dried overnight at 95 °C to remove moisture and obtain a dry cobalt hydroxide precursor. To achieve the desired crystalline Co_3_O_4_ phase, the dried powder was calcined in a muffle furnace at 600 °C. This calcination step decomposed the hydroxide precursor and resulted in the formation of crystalline cobalt oxide nanoparticles. The final Co_3_O_4_ powder was collected for subsequent structural and electrochemical characterization.

### 2.3. Synthesis of MXene

The etching of the aluminum layer in the MAX phase was performed using an oxidizing-ligand system comprising hydrochloric acid (HCl) and lithium fluoride (LiF). A 9 M HCl solution was prepared by diluting concentrated HCl with distilled water. Lithium fluoride (0.75 g) was then added to the HCl solution and stirred at 50 °C for 2 h to form a homogeneous acidic solution containing reactive H^+^ and F^−^ species. Subsequently, 0.325 g of Ti_3_AlC_2_ MAX phase powder was gradually introduced over 30 min to control the exothermic reaction and prevent agglomeration. The mixture was reacted for 48 h to ensure complete oxidation of the aluminum layer to Al^3+^ and its conversion to AlF_3_. After the reaction, the product was centrifuged and washed repeatedly with distilled water and ethanol until the supernatant reached a pH ≥ 5.5, removing residual impurities. The sediment was then ultrasonicated for 1 h to delaminate the MXene sheets and dried overnight at 60 °C under vacuum to yield the final MXene material, and air exposure was minimized during electrode preparation.

### 2.4. Synthesis of Co_3_O_4_/MXene Composite

The Co_3_O_4_/MXene composite was prepared at different weight ratios by dispersing Co_3_O_4_ powder in 50 mL of distilled water, followed by 1 h of ultrasonication under continuous water cooling to ensure homogeneity. Subsequently, 10 mg of MXene according to ratio was added to the dispersion, and the mixture underwent an additional 2 h of ultrasonication to facilitate Co_3_O_4_ intercalation within the MXene layers. The product was then centrifuged at 3500 rpm and washed with distilled water and ethanol to remove residual impurities. Finally, the composite was dried overnight at 60 °C to yield the final Co_3_O_4_/MXene material.

### 2.5. Co_3_O_4_/MXene Composite Electrode Fabrication

The working electrode suspension was prepared by dispersing 2.0 mg of Co_3_O_4_/MXene nanocomposite in 1 mL of 2% Nafion solution. The mixture was sonicated for 1 h to achieve a homogeneous suspension of the Co_3_O_4_/MXene composite. Subsequently, a 10 µL aliquot of the suspension was drop-cast onto the working electrode and dried at 50 °C for 2 h to obtain the modified working electrode.

### 2.6. Electrochemical Measurements

An electrochemical workstation was used to perform cyclic voltammetry (CV), galvanostatic charge–discharge (GCD), and electrochemical impedance spectroscopy (EIS) of the Co_3_O_4_ and Co_3_O_4_/MXene composites. Further, the scan rate test was conducted in a potential range of 10 to 50 mV s^−1^, and GCD studies were conducted at a current density range from 1 to 5 mA g^−1^, respectively. Electrochemical measurements were conducted using 2 M KOH, Hg/HgO, and a three electrode system such as platinum wire, reference electrode, and a counter electrode, respectively. The fabricated Co_3_O_4_ and Co_3_O_4_/MXene composite electrodes were used as working electrodes for examining their electrochemical responses. The specific capacitance of the fabricated material was calculated from both the CV and GCD data using.

(1) $$Csp=\frac{Area}{2\times ∆V\times m\times scan\;rate}\;$$

(2) $$Csp=\frac{I\times t}{m\times ∆V}$$

C_sp,_ represent the specific capacitance that can be calculated from CV and GCD data (F g^−1^), potential window (∆V), which was 0.6 V for the current study, varied scan rate, current at which the GCD was run (I), discharge time (t), mass loaded (m), respectively.

### 2.7. Characterization Techniques

Characterization of the synthesized materials was conducted at the University-Wide Range Research Facilities (CUWRF), Jeonbuk National University (JBNU), South Korea. Crystallographic data were acquired using X-ray diffraction (XRD-6100 (Shimadzu, Kyoto, Japan), Cu Kα radiation, λ = 0.15418 nm). Surface morphology was observed with a JSM-5400 scanning electron microscope (JEOL, Seoul, South Korea). Functional groups were identified through Fourier-transform infrared (FTIR) spectroscopy (Perkin Elmer, Frontier, Paju, South Korea). Morphological and elemental analysis was performed via field-emission scanning electron microscopy (FESEM, SUPRA40VP, Seoul, South Korea), which included energy-dispersive spectroscopy (EDS) for compositional insights.

## 3. Characterization Analysis

### 3.1. XRD Analysis

**Figure 1:**
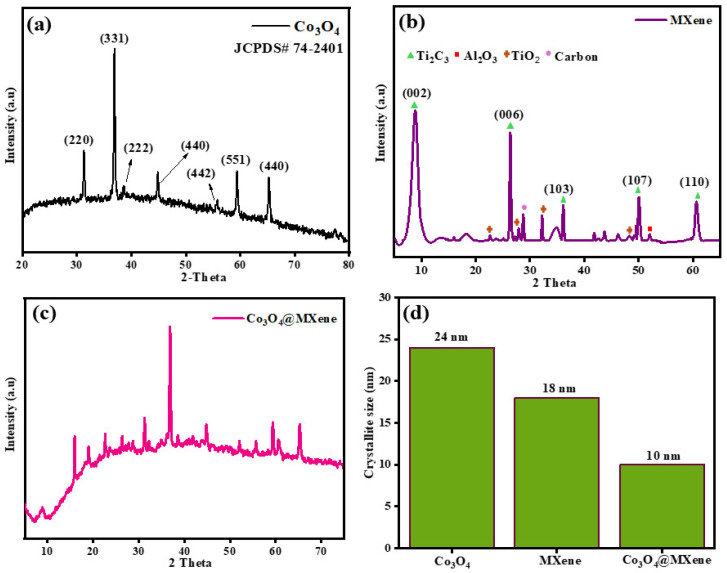
X-ray diffraction patterns of (**a**) Co_3_O_4_, (**b**) MXene (**c**) Co_3_O_4_@ MXene composite (**d**) crystallite size of Co_3_O_4_@ MXene, Co_3_O_4_, and MXene.

XRD patterns of pristine cobalt oxide, MXene, and the Co_3_O_4_@MXene composite are shown in Figure 1. The diffraction profile labeled as Co_3_O_4_ displays sharp reflections at approximately 2θ ≈ 31.2°, 36.8°, 44.8°, 55.6°, 59.3°, and 65.2°. These peaks are well indexed to the cubic spinel Co_3_O_4_ phase (JCPDS card no. 74-1657), corresponding to the (220), (311), (400), (422), (511), and (440) planes, respectively [21]. Thus, the hydrothermal precursor followed by annealing likely produced crystalline Co_3_O_4_ [22]. The MXene (Ti_3_C_2_T_x_) pattern shows a broad reflection in the low-angle region (<10°), which is outside the displayed scan window, but higher-angle features are visible around 18–19°, 27–28°, and 60–61°. These peaks are associated with (002) reflections and in-plane (110) reflections of Ti_3_C_2_T_x_ MXene [23]. The multiple small peaks visible in the MXene profile suggest partial restacking and the presence of surface functional groups. The calculated d_002_ spacing and peak broadening indicate mild intercalation of Co ions rather than oxidation. Few-layer stacking was observed, not fully single-layer colloids, which explains a moderate (002) intensity and some restacking. No anatase or rutile peaks; (002) remains dominant at low angle with expected d spacing, suggesting Ti_3_C_2_T_x_ retained its layered structure with minimal oxidation.

In the Co_3_O_4_@MXene composite, the diffraction peaks of both phases are retained, confirming successful hybridization without secondary phase formation such as cobalt titanates. Notably, the Co-oxide peaks are significantly broadened relative to pristine Co-oxide, implying a reduction in coherent domain size when the oxide nucleates on the MXene substrate. The bar chart included in Figure 1d summarizes crystallite size estimations obtained by the Scherrer equation. The average crystallite size of the pristine Co-oxide was ~24 nm, which decreased to ~18 nm for MXene domains and further to ~10 nm for the Co_3_O_4_@MXene composite. This reduction in crystallite size is consistent with MXene sheets restricting oxide grain growth during nucleation. Smaller crystallites are advantageous for supercapacitor applications, as they shorten ion diffusion pathways, increase electroactive surface area, and improve the kinetics of redox processes [24]. Overall, the XRD analysis confirms that the synthesized material is a Co-oxide@MXene hybrid, with the oxide phase matching spinel Co_3_O_4_. The intimate integration with MXene nanosheets suppresses particle growth and enhances the structural features favorable for charge storage.

### 3.2. FTIR Spectroscopic Analysis

**Figure 2:**
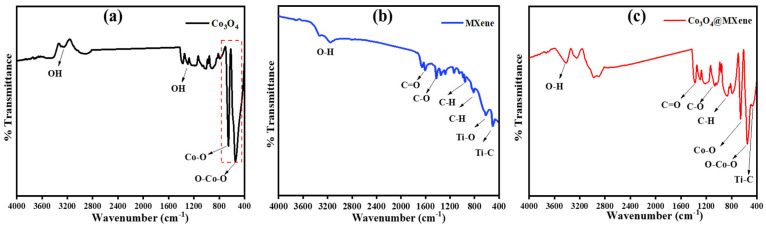
FTIR spectra of (**a**) Co_3_O_4_, (**b**) MXene, (**c**) Co_3_O_4_@ MXene nanomaterials.

FTIR spectroscopy was carried out to investigate the functional groups and bonding interactions in pristine Co_3_O_4_, MXene, and the Co_3_O_4_@MXene composite, as represented in Figure 2. For Co_3_O_4_, a prominent absorption band is observed around ~560–580 cm^−1^, which corresponds to the stretching vibrations of Co–O bonds, confirming the formation of cobalt oxide [25]. Additionally, weak features in the 3400 cm^−1^ region are assigned to adsorbed hydroxyl groups (O–H stretching), which are commonly present due to surface hydration. For MXene (Ti_3_C_2_T_x_), characteristic peaks are observed at ~3400 cm^−1^ and ~1630 cm^−1^, assigned to O–H stretching and bending vibrations of surface hydroxyl groups, respectively [26]. Peaks in the region 500–700 cm^−1^ correspond to Ti–C and Ti–O vibrations, confirming the presence of functionalized MXene sheets [27]. The broad absorptions between 1000 and 1400 cm^−1^ are related to C–O and C=O surface groups, which originate from surface terminations introduced during the etching process [28]. For the Co_3_O_4_@MXene composite, the FTIR spectrum displays features of both parent materials, indicating successful integration of Co_3_O_4_ with MXene nanosheets. The Co–O vibration is clearly visible along with Ti–O/Ti–C modes, demonstrating coexistence of both phases. Interestingly, the O–H and C=O related bands are shifted and broadened compared to the pristine samples, suggesting strong interfacial interactions through hydrogen bonding and electrostatic interactions between the Co_3_O_4_ nanoparticles and the functional groups on MXene surfaces, showing successful structural arrangements [29]. This interaction likely enhances the stability of the composite and facilitates electron/ion transport at the interface. FTIR analysis confirms that Co_3_O_4_ nanoparticles are successfully anchored onto MXene sheets without destroying the structural integrity of either component. The observed shifts in vibrational bands provide strong evidence of chemical interactions, which are expected to play a key role in improving the electrochemical performance of Co_3_O_4_@MXene electrode material owing to improved charge transfer and energy storage.

### 3.3. Morphological Analysis

**Figure 3:**
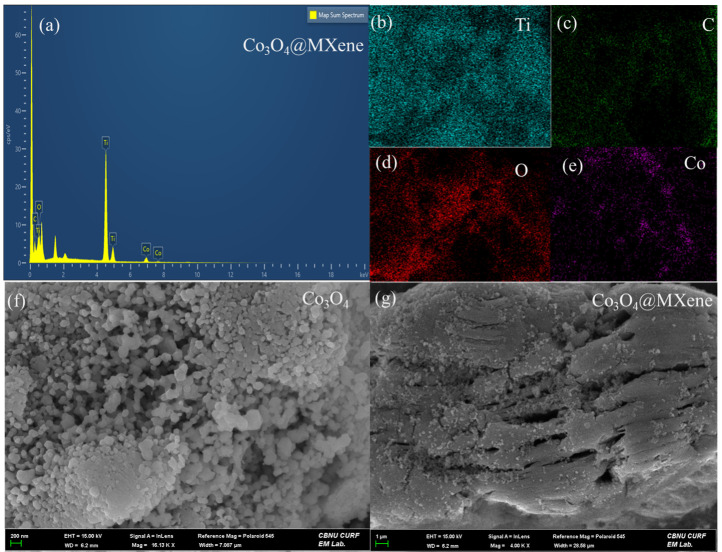
(**a**) EDX of Co_3_O_4_@ MXene, (**b**–**e**) electronic mapping of Ti, C, O, Co from Co_3_O_4_@ MXene nanomaterials following EDX (**f**) SEM of Co_3_O_4_ (**g**) Co_3_O_4_@ MXene nanomaterials.

The elemental and morphological characterization of the Co_3_O_4_@MXene composite as shown in Figure 3 reveals crucial insights into its compositional uniformity and microstructural features. The energy-dispersive X-ray spectroscopy (EDS) spectrum confirms the presence of titanium (Ti) and carbon (C) from the MXene, alongside cobalt (Co) and oxygen (O) originating from the cobalt oxide nanoparticles. This elemental composition affirms the successful synthesis of the composite material. Elemental mapping further supports this finding, showing a uniform distribution of Ti and C consistent with the MXene’s layered structure, while Co and O are homogeneously dispersed, indicating well-distributed Co_3_O_4_ nanoparticles on the MXene sheets. The distinct spatial co-localization of cobalt and oxygen responses corroborates the effective loading of cobalt oxide onto the conductive MXene substrate [30].

Morphological analysis through scanning electron microscopy (SEM) depicts the pristine MXene and the Co_3_O_4_@MXene composite surface that were used in electrochemical applications. The pristine MXene exhibits a typical stacked and layered nanosheet morphology with relatively smooth surfaces and tightly packed layers. In the composite, the surface topology changes distinctly, with coarse granules representing Co_3_O_4_ nanoparticles distributed across the MXene layers. This enhanced roughness and the appearance of cracks and interlayer gaps promote better electrolyte penetration and ion accessibility, which are advantageous for electrochemical processes such as pseudocapacitive charge storage in supercapacitors. These combined structural and elemental features reflect the synergistic effect where the highly conductive MXene scaffold supports the redox-active Co_3_O_4_ particles, leading to improved charge transfer and electrochemical performance [31].

### 3.4. Cyclic Voltammetry

**Figure 4:**
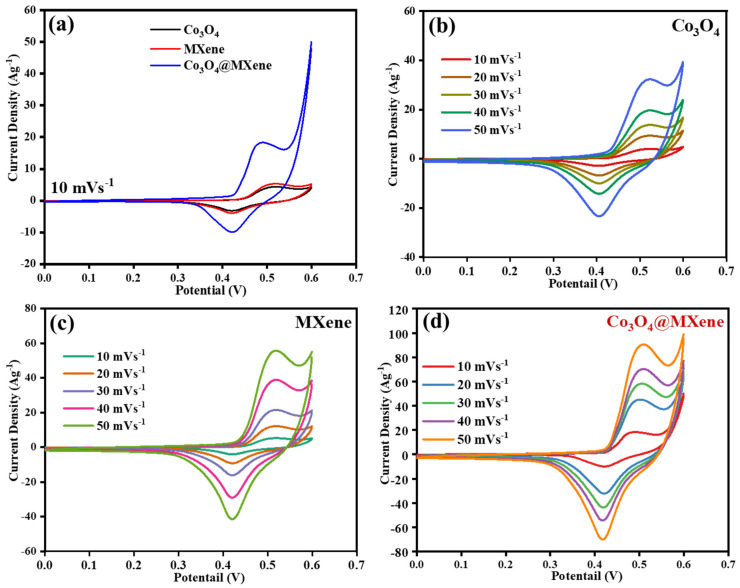
Cyclic voltammetry of: (**a**) Co_3_O_4_@ MXene, Co_3_O_4_ and MXene at 10 mVs^−1^, CV profiles of (**b**) Co_3_O_4_ (**c**) MXene and (**d**) Co_3_O_4_@ MXene at 10–50 mVs^−1^.

Figure 4 compares the CV responses of pristine oxide, pristine MXene, and the hybrid Co-oxide@MXene over scan rates from 10 to 50 mV s^−1^ within the same potential window. The trace labeled Co oxide exhibits clear anodic and cathodic peaks that grow with scan rate, consistent with Faradaic pseudocapacitance arising from cobalt redox couples in alkaline media. Two surface-confined couples are typically observed for spinel Co_3_O_4_: Co_3_O_4_Co-OOH at lower potential and Co-OOH/CoO_2_ at higher potential, both quasi-reversible under supercapacitor conditions. Their midpoint potentials reported using 2 M KOH vs. Hg/HgO are near 0.29 V and 0.56–0.60 V, respectively, which matches the peak positions commonly seen for Co_3_O_4_ electrodes and explains the pronounced humps in oxide CVs. Peak separation increases somewhat with scan rate, which is expected due to kinetic polarization and uncompensated resistance [32]. The MXene (Ti_3_C_2_T_x_) CVs show near-rectangular profiles that scale almost linearly with scan rate, characteristic of predominantly electric double-layer charge storage with minor surface redox from terminations or intercalation. Ti_3_C_2_T_x_ is metallically conductive and hydrophilic, so ions access interlayer galleries rapidly, and the current response remains close to ideal capacitive behavior even at higher scan rates. This is consistent with seminal MXene studies that reported high-rate rectangular CVs and large volumetric capacitance due to rapid cation intercalation between Ti_3_C_2_ layers [14].

The composite Co-oxide@MXene combines features of both parents. Its CVs retain the redox peaks of cobalt oxide, but the overall enclosed area and peak currents are larger than either single component at the same scan rate, indicating a higher total stored charge. At low scan rates the peaks are relatively sharp, which suggests facile charge transfer at the oxide–MXene interface. At higher scan rates, the composite maintains its peak shape more effectively than the oxide alone, implying better rate capability. These improvements are consistent with a synergistic mechanism in which the MXene network provides fast electron pathways and short ion diffusion lengths while the nano dispersed cobalt oxide domains supply redox-active sites. Similar enhancements have been reported widely for MXene–metal-oxide hybrids used as supercapacitor electrodes [33]. From a kinetics viewpoint, the qualitative trends across scan rate indicate mixed storage: a surface-controlled capacitive component that scales with v and a diffusion-controlled Faradaic component that scales with v^1/2^ [34].

Overall, the CV evidence indicates the following: (i) pristine oxide stores charge through Co-centered pseudocapacitive reactions but suffers increasing polarization at high rates, (ii) pristine MXene delivers rapid, mostly double-layer charge storage with modest pseudocapacitance, and (iii) the Co-oxide@MXene hybrid integrates both behaviors, achieving larger charge and better retention of redox features at high scan rates due to interfacial synergy between the redox-active oxide and the highly conductive, ion-permeable MXene network. These conclusions align with the established understanding of Co-oxide electrochemistry in alkaline electrolytes and of MXene-based supercapacitors.

### 3.5. Capacitive vs. Diffusive Contribution

As current density rises, the discharge time Δt shortens in Csp = I·Δt/(m·ΔV), so the apparent capacitance drops because slow diffusion-limited Faradaic reactions cannot fully contribute at high rates. This is exactly what one expects for oxide or MXene electrodes under galvanostatic testing and is consistent with standard supercapacitor methodology [35].

**Figure 5:**
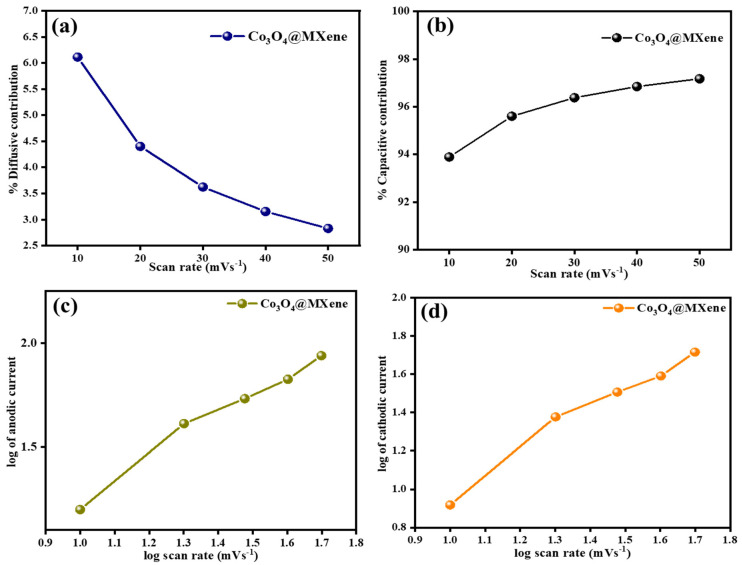
(**a**) Diffusive contributions (**b**) capacitive contribution, (**c**) Dunn plot for anodic peak current (**d**) Dunn plot of cathodic peak current for Co_3_O_4_@ MXene nanomaterials.

As shown in Figure 5, the increasing Co_3_O_4_@Mxene anodic and cathodic curves are characteristic of IR drop, ΔVIR, or ESR-related metrics versus current density, since ΔVIR ≈ I·RESR increases nearly linearly with I. The lower slope corresponds to lower ESR and better rate performance. A lower IR drop is typically observed when a redox-active phase is wired by a highly conductive scaffold, which is the intended role of Ti_3_C_2_Tx in the Co-oxide@MXene hybrid [36]. A monotonic increase with scan rate usually indicates a larger surface-controlled contribution at higher rates. That interpretation is fully consistent with the CV set: pristine MXene exhibits nearly rectangular, scan-rate-proportional CVs dominated by double-layer storage, while the composite retains the redox humps of cobalt oxide yet encloses a larger overall CV area and keeps the peaks more visible at high rate because the MXene network accelerates charge transfer [14].

### 3.6. Galvanostatic Charge–Discharge

**Figure 6:**
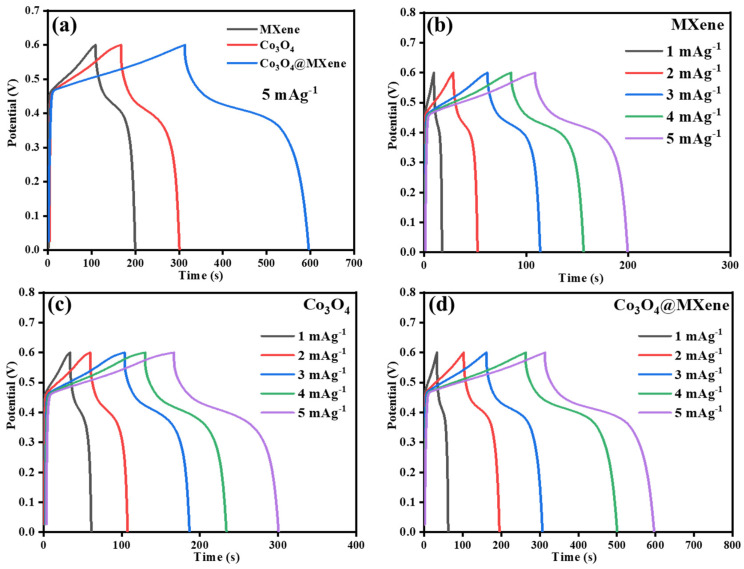
Galvanostatic charging/discharging curves of: (**a**) Co_3_O_4_@ MXene, Co_3_O_4_ and MXene at 5 mAg^−1^, GCD profiles of (**b**) MXene (**c**) Co_3_O_4_ and (**d**) Co_3_O_4_@ MXene at current density of 1–5 mAg^−1^.

The galvanostatic charge–discharge (GCD) profiles of pristine Co_3_O_4_, pristine MXene, and the Co_3_O_4_@MXene composite at different current densities are shown in Figure 6. All electrodes display nearly symmetric charge and discharge traces, indicating high Coulombic efficiency and good reversibility of the charge storage process. The pristine MXene electrode exhibits almost triangular GCD curves, typical of electric double-layer capacitance with minor pseudocapacitive contributions from surface terminations [37]. In contrast, the Co_3_O_4_ electrode shows nonlinear discharge curves with clear deviations from triangular shape, reflecting the Faradaic redox reactions associated with Co^2+^/Co^3+^ and Co^3+^/Co^4+^ couples, which are well-known for cobalt oxides in alkaline electrolytes. The composite electrode combines these two behaviors, showing extended discharge times along with pseudocapacitive features, suggesting synergistic contributions from both components [38].

At lower current densities (e.g., 1 A g^−1^), the discharge duration of the composite is significantly longer than that of pristine Co_3_O_4_ or MXene, corresponding to its higher specific capacitance. As the current density increases, all electrodes exhibit shorter discharge times due to kinetic and diffusion limitations, yet the composite retains much higher capacitance compared with the individual materials, reflecting excellent rate capability. This observation is consistent with the CV results, where the Co_3_O_4_@MXene electrode preserved pronounced redox peaks and a larger integrated area even at higher scan rates. The IR drop behavior also highlights the advantage of the composite: pristine Co_3_O_4_ displays a pronounced IR drop due to its poor conductivity, while MXene shows negligible IR drop but lower total capacitance. The hybrid electrode exhibits both a reduced IR drop and an extended discharge time, confirming that the MXene framework effectively lowers resistance and enhances ion/electron transport at the oxide–MXene interface.

### 3.7. Electrochemical Impedance Spectroscopy

**Figure 7:**
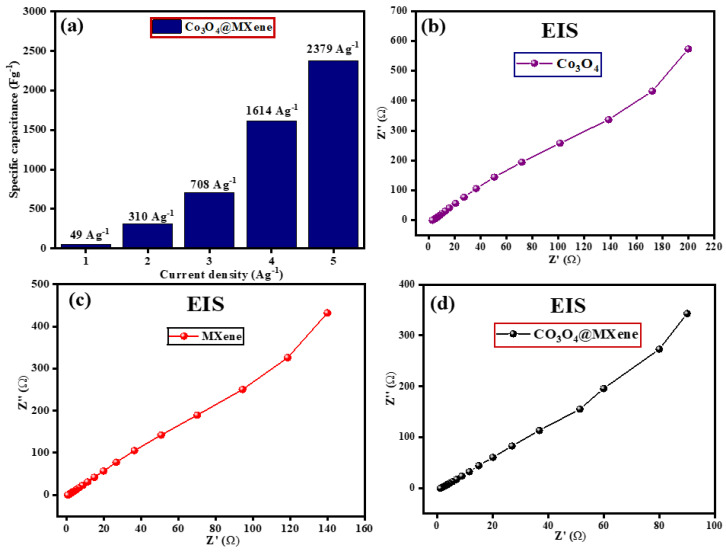
(**a**) Specific capacitance of Co_3_O_4_@ MXene at current density of 1–5 mAg^−1^. EIS profiles of (**b**) Co_3_O_4_, (**c**) MXene and (**d**) Co_3_O_4_@ MXene nanomaterials.

Figure 7 presents a comprehensive comparison of supercapacitive performance between pristine Co_3_O_4_ and the Co_3_O_4_@MXene composite using specific capacitance-current density analysis and electrochemical impedance spectroscopy (EIS). Figure 7a depicts the specific capacitance values of Co_3_O_4_@MXene as a function of increasing current density. The composite yields a notably low capacitance of 49 F g^−1^ at 1 A g^−1^, which rapidly increases to 310, 708, 1614, and finally 2379 F g^−1^ at 5 A g^−1^. This exceptional trend demonstrates the material’s ability to store charge efficiently even at high charging rates, an unusual feature since capacitance typically drops at higher current densities due to limited ion transport and insufficient time for redox reactions. The observed phenomenon indicates rapid electron/ion transfer and reversible faradaic activity, attributed to the synergistic effect between the conductive MXene layers and the pseudocapacitive behavior of Co_3_O_4_ [39].

Figure 7b,c show the EIS Nyquist plots for pristine Co_3_O_4_ and Co_3_O_4_@MXene, respectively. In panel (b), Co_3_O_4_ displays a pronounced semicircle and higher impedance values, revealing substantial charge-transfer resistance and moderate ion-diffusion properties. In contrast, panel (c) demonstrates that the Co_3_O_4_@MXene composite has significantly reduced impedance and a much smaller semicircle, suggesting a lower internal resistance and faster charge-transfer kinetics. This impedance reduction is logical, given the two-dimensional MXene framework enhances electronic conductivity and facilitates ion transport across the composite structure. Taken together, these data highlight the superior electrochemical performance of Co_3_O_4_@MXene over pure Co_3_O_4_, featuring high specific capacitance at elevated current densities and improved charge-transfer properties. These attributes are crucial for advanced supercapacitor electrodes, ensuring both high energy storage capability and rapid response during charging and discharging cycles.

These kinetic conclusions are consistent with earlier datasets. FTIR confirmed that both Co–O and Ti–C/Ti–O groups coexist in the composite, which supports intimate interfacial contact and good wettability. XRD showed that the oxide pattern matches spinel Co_3_O_4_ and that peaks are broader in the composite, implying smaller coherent domains; smaller crystallites shorten diffusion paths and increase the density of electroactive sites. CV already displayed pronounced cobalt redox peaks for the oxide, rectangular profiles for MXene, and a larger enclosed area with preserved peaks for the composite across 10–50 mV s^−1^, which is exactly what a higher b value and a larger capacitive fraction anticipate. GCD curves then translated this into practice: the composite delivered the longest discharge times at a given current, retained capacitance better as current increased, and showed a reduced IR drop, meaning a lower effective series resistance. All four figure panels here are therefore a kinetic corollary to the structural and electrochemical evidence: the Co-oxide@MXene interface suppresses oxide coarsening, accelerates electron and ion transport, and shifts the operative mechanism toward fast, surface-controlled storage while keeping the Faradaic energy of cobalt oxide.

## 4. Conclusions

The Co_3_O_4_@MXene nanocomposite synthesized in this study demonstrates a remarkable integration of cobalt oxide’s outstanding pseudocapacitive properties with the exceptional electrical conductivity and mechanical flexibility of MXene nanosheets. Structural characterization confirms the successful hybridization, where nanoscale Co_3_O_4_ particles are uniformly anchored onto the MXene substrate without compromising the intrinsic structural integrity of either component. This intimate interfacial contact not only prevents particle agglomeration but also facilitates rapid electron and ion transport, reducing charge-transfer resistance substantially. The hybrid electrode delivers an ultrahigh specific capacitance of 2379 F g^−1^ at 1 A g^−1^, which surpasses pristine Co_3_O_4_ and MXene electrodes by a significant margin. Furthermore, the Co_3_O_4_@MXene exhibits superior rate capability, retaining excellent capacitance retention at high current densities, a testament to its efficient charge storage kinetics. These findings highlight the potential of Co_3_O_4_@MXene as a robust electrode for high-power supercapacitors, combining fast kinetics with long-term stability.

## Data Availability

The data presented in this study are available on request from the corresponding author.
